# Expression of Programmed Death-Ligand 1 by Human Colonic CD90^+^ Stromal Cells Differs Between Ulcerative Colitis and Crohn’s Disease and Determines Their Capacity to Suppress Th1 Cells

**DOI:** 10.3389/fimmu.2018.01125

**Published:** 2018-05-30

**Authors:** Ellen J. Beswick, Carl Grim, Abinav Singh, Jose E. Aguirre, Marissa Tafoya, Suimin Qiu, Gerhard Rogler, Rohini McKee, Von Samedi, Thomas Y. Ma, Victor E. Reyes, Don W. Powell, Irina V. Pinchuk

**Affiliations:** ^1^Department of Molecular Genetics and Microbiology, University of New Mexico, Albuquerque, NM, United States; ^2^Department of Internal Medicine, University of Texas Medical Branch, Galveston, TX, United States; ^3^Department of Pathology, University of New Mexico, Albuquerque, NM, United States; ^4^Department of Pathology, University of Texas Medical Branch, Galveston, TX, United States; ^5^Department of Gastroenterology and Hepatology, University Hospital of Zürich, Zürich, Switzerland; ^6^Department of Surgery, University of New Mexico, Albuquerque, NM, United States; ^7^Department of Internal Medicine, Division of Gastroenterology and Hepatology, University of New Mexico, Albuquerque, NM, United States; ^8^Department of Microbiology and Immunology, University of Texas Medical Branch, Galveston, TX, United States; ^9^Department of Pediatrics, University of Texas Medical Branch, Galveston, TX, United States

**Keywords:** myofibroblasts, fibroblasts, programmed death-ligand 1, PD-L2, ulcerative colitis, Crohn’s disease, inflammatory bowel diseases, Th1 cells

## Abstract

**Background and Aims:**

The role of programmed cell death protein 1 (PD-1) and its ligands in the dysregulation of T helper immune responses observed in the inflammatory bowel disease (IBD) is unclear. Recently, a novel concept emerged that CD90^+^ colonic (myo)fibroblasts (CMFs), also known as stromal cells, act as immunosuppressors, and are among the key regulators of acute and chronic inflammation. The objective of this study was to determine if the level of the PD-1 ligands is changed in the IBD inflamed colonic mucosa and to test the hypothesis that changes in IBD-CMF-mediated PD-1 ligand-linked immunosuppression is a mechanism promoting the dysregulation of Th1 cell responses.

**Methods:**

Tissues and cells derived from Crohn’s disease (CD), ulcerative colitis (UC), and healthy individuals (N) were studied *in situ, ex vivo*, and in culture.

**Results:**

A significant increase in programmed death-ligand 1 (PD-L1) was observed in the inflamed UC colonic mucosa when compared to the non-inflamed matched tissue samples, CD, and healthy controls. UC-CMFs were among the major populations in the colonic mucosa contributing to the enhanced PD-L1 expression. In contrast, PD-L1 expression was decreased in CD-CMFs. When compared to CD-CMFs and N-CMFs, UC-CMFs demonstrated stronger suppression of IL-2, Th1 transcriptional factor Tbet, and IFN-γ expression by CD3/CD28-activated CD4^+^ T cells, and this process was PD-L1 dependent. Similar observations were made when differentiated Th1 cells were cocultured with UC-CMFs. In contrast, CD-CMFs showed reduced capacity to suppress Th1 cell activity and addition of recombinant PD-L1 Fc to CD-CMF:T cell cocultures partially restored the suppression of the Th1 type responses.

**Conclusion:**

We present evidence showing that increased PD-L1 expression suppresses Th1 cell activity in UC. In contrast, loss of PD-L1 expression observed in CD contributes to the persistence of the Th1 inflammatory milieu in CD. Our data suggest that dysregulation of the Th1 responses in the inflamed colonic mucosa of IBD patients is promoted by the alterations in PD-L1 expression in the mucosal mesenchymal stromal cell compartment.

## Introduction

Dysregulation of T helper (Th) cell responses in inflammatory bowel disease (IBD) is critically involved in colonic tissue damage. Crohn’s disease (CD) is characterized by elevation of Th1 cells that express the Tbet transcription factor and produce IFN-γ (1). In contrast, for ulcerative colitis (UC) colonic mucosal production of IFN-γ is either suppressed or comparable to normal tissue with an increase in Th2 cytokines IL-5 and IL-13 (2–4). While this disruption of balanced Th1/Th2 cell responses is among the important effector mechanisms contributing to the IBD pathophysiology, the mechanisms responsible for this dysregulation remain unclear.

Innate immune cells are important regulators of the T cell responses in the colon. TLR-dependent activation of dendritic cells and macrophages contribute to the dysregulation of T cell responses in IBD *via* increased production of inflammatory cytokines, such as TNF-α, IL-12, and IL-23 (5–8). In addition to the inflammatory cytokines, the B7 family of proteins also regulates T cell responses (9, 10). Interactions of the B7 molecules programmed death-ligand 1 (PD-L1) and/or PD-L2 with programmed cell death protein 1 (PD-1) are known to control several tolerance checkpoints that prevent autoimmunity (11). Abnormities in PD-L1 and PD-L2 expression/signaling contribute to several chronic infectious and inflammatory diseases, such as type 1 diabetes, rheumatoid arthritis, allergy, and chronic obstructive pulmonary disease. In these diseases, alterations in the expression and signaling of PD-1 and its ligands result in the dysregulation of Th1/Th2 responses and overall IFNγ production (11–15).

Programmed death-ligand 1- and PD-L2-mediated signaling by innate immune cells is crucial to the maintenance of the mucosal tolerance in the GI tract (16–18). Loss of PD-L1 signaling in the gut breaks CD8^+^ T cell tolerance to self-antigens and leads to severe autoimmune enteritis (18). The few reports on the role of PD-1 and its ligands in murine models of chronic colitis remain contradictory. PD-1 deficiency impairs induction of regulatory T cells and promotes severe CD-like colitis (19), while PD-L1 expression by DX5^+^NKT cells induces apoptosis of colitic CD4^+^ T cells (20). Suppression of PD-L1 with anti-PD-L1 monoclonal antibodies (mAbs) reduced chronic intestinal inflammation in the T cell transfer murine model of colitis (21), whereas use of a PD-L1Fc was shown to protect against T cell transfer-colitis (22). Furthermore, there is a worsening of DSS and TNBS acute colitis in PD-L1^−/−^ mice compared to wild-type animals (23).

The intricate role of PD-L1 and PD-L2 in the dysregulation of Th cell responses in human IBD remains unclear and the sparse reports are contradictory. PD-L1 is upregulated in the intestinal epithelium, macrophages, and B cells in both forms of IBD (21, 24), yet expression of inducible PD-L1 appears to be impaired in CD-derived monocytes and ileal APCs (25, 26). Finally, recent reports indicate that while mAbs against PD-1 and anti-PD-L1 are currently successfully used in clinics for treatment of several solid tumors, one of the main immune-related adverse effect (irAE) of the immune checkpoint blockade therapy is development of chronic diarrhea and enterocolitis (27–29). A recent case report describes Crohn’s colitis-like phenotype as an irAE (30). Therefore, the role of these molecules in several types of IBD colitis warrants further investigation.

We previously reported that in the normal colonic mucosa, CD90^+^ stromal cells, otherwise known as colonic (myo)fibroblasts (CMFs) are a major cell type expressing PD-L1 and PD-L2 (16). CMFs act as major immunosuppressors under mucosal tolerance (16, 31, 32) and both molecules have been implicated in normal CMF-mediated suppression of activated CD4^+^ T cell proliferation (16). Normal CMFs suppress IFN-γ production by Th cells *via* PD-L1-mediated mechanism (32), but PD-L1/PD-L2 signaling is poorly characterized in IBD. Thus, PD-1 ligand signaling in IBD and in other types of colitis such as that associated with checkpoint immunotherapy of cancer warrants more investigation.

In this report, we evaluated PD-L1 and PDL-2 expression in human IBD colonic mucosa and tested the hypothesis that changes in PD-1 ligand-mediated CMF signaling contributes to the dysregulation of Th1/Th2 cell responses in human IBD. We demonstrated that compared to normal or IBD non-inflamed colonic mucosa PD-L1, but not PD-L2, was strongly increased in UC and somewhat decreased in CD. We observed that PD-L1 is critical to the CMF-mediated regulation of the Th1 cell cytokine production. Further, we found that increase in PD-L1 by UC-derived CMFs contribute to the increased suppression of Th1 cell activity. In contrast, lower expression of PD-L1 by CD-CMFs contributed to the increase in the Th1 cell responses observed in CD. Taken together, our data identify CMFs as an important immunological component in colonic mucosa and suggest that changes in the CMF-mediated PD-L1 expression may be critical to the pathological dysregulation of the Th1 immune responses in IBD.

## Materials and Methods

### Antibodies

Fluorochrome-conjugated and unconjugated murine anti-human α-smooth muscle actin (α-SMA, clone 1A4) monoclonal antibodies (mAbs) were purchased from Sigma (St. Louis, MO, USA). Fluorochrome-conjugated forms of IgG1_κ_, IgG_2a_, isotype controls, and mAbs directed against human CD90 (clone 5E10) were purchased from BD Bioscience and eBioscience (San Diego, CA, USA). Fluorochrome-conjugated antibodies against human and murine CD4 (clone RPA-T4 and RM4-5, respectively), Tbet (clone eBIo4B10), isotype controls as well as mAbs against human PD ligands, PD-L1 (clone MIH1, clone 29E.2A3), and PD-L2 (clone MIH18), antihuman IFN-γ (clone 45.B3) were obtained from eBioscience (San Diego, CA, USA) and BioLegend Inc., Alexa Fluor^®^ (AF^®^) 488- and AF^®^633-labeled donkey anti-mouse IgG_2a_ and IgG1_κ_ (respectively), Zenon Mouse IgG antibodies labeling kits were purchased from Life Science Technology, Inc. (CA, USA). Functional grade anti-human IL-4 mAbs (clone B-S4), recombinant human IL-12 and IL-2 were purchased from ThermoFisher Scientific.

### Tissue and Cells

For CMF isolation, full thickness fresh human colonic mucosa samples were obtained from discarded surgical resections under UTMB and UNM Health Sciences Center approved IRB protocols #99-061 and #10-514, respectively. Surgical resection tissues from patients undergoing colectomy for UC, CD, or colon cancer (only normal margin used) were obtained. As discarded tissue, their use did not require informed consent, Endoscopic colonic biopsies were obtained after subject informed consent under UTMB approved human research protocol IRB# 03-392. Vulnerable populations (e.g., age less than 18) were not included in study. The information on the tissue samples used for cell isolation in this study is provided in the Table 1. Total mucosal cell (CM) preparation was done as described previously (33). CMFs were isolated as previously described (32, 34). Studies were performed with primary isolates of CMFs at passages 3–10. Standard MEM (ThermoFisher) + 10% FBS, 2 mM l-glutamine, 100 U/ml penicillin, 100 g/ml streptomycin, 1 mM sodium pyruvate was used for growth of both, CMF and T cells.

**Table 1:** Patient characteristics.

	Normal, control, *N* = 11	UC^a^, *N* = 21	CD^a^, *N* = 22
**Age**			
A2(18–40)	2 (18%)	9 (43%)	10 (45%)
A3 (≥40)	9 (82%)	12 (57%)	12 (55%)
**Sex**			
Male	5 (45%)	12 (57%)	12 (55%)
Female	6 (55%)	9 (43%)	10 (45%)
**Location**			
Ileum and cecum			9 (48%)
Right colon	2 (18%)	1 (5%)	1 (4%)
Transverse colon	3 (27%)		1 (4%)
Left colon	2 (18%)	2 (10%)	2 (8%)
Sigmoid/rectum	4 (37%)	5 (25%)	4 (16%)
Colon, mixed		13 (60%)	5 (20%)
**Therapy**			
5-ASA	n/a	3 (14%)	0
Corticosteroids	n/a	2 (5%)	0
Immunomodulators/corticosteroids	n/a	3 (14%)	0
5-ASA/corticosteroids	n/a	0 (0%)	6 (27%)
Biologics	n/a	0 (0%)	7 (32%)
Mix of biologics with corticosteroids/immunomodulators	n/a	8 (43%)	5 (23%)
No treatment	11 (100%)	5 (24%)	4 (18%)

*^a^All patients included in this study had active disease, tissue inflammation was scored as moderate/severe*.

### Confocal Microscopy

Frozen human colonic step sections were fixed and immunostained as described previously (16). Briefly, OCT frozen human colonic tissue sections were fixed in 1% paraformaldehyde for 20 min at room temperature, blocked with a PBS based blocking solution containing 2.5% normal murine serum and 1 µg/ml of human BD Fc Block™ reagent (BD Bioscience, San Jose, CA, USA) for 15 min at room temperature. Unless otherwise indicated, samples were then incubated with AF546-conjugated anti–PD-L1 (clone MIH1) and AF488-conjugated anti-human CD90 (clone 5E10), anti-human α-SMA mAbs (clone 1A1) or AF647-conjugated anti-human EpCAM mAbs, or appropriate fluorochrome-conjugated mouse IgG1κ isotype control for 2 h at room temperature. Six time wash with Ca^++^Mg^++^PBS was performed before and after of the each of the described above steps. Samples were then mounted in SlowFade^®^ Gold antifade reagent with DAPI (Life Science Technology, CA, USA). Confocal microscopy was performed with a Zeiss LSM510 META laser scanning confocal microscope (Carl Zeiss, Thornwood, NY, USA) (16). To quantify expression of the molecule of interest, the integrated density, area, and mean of fluorescence from at least at least 10 fields of in-focus images from each tissue were measured using ImageJ software (NIH, https://imagej.nih.gov/ij/). The Corrected Total Cell Fluorescence (CTCF) was then calculated according the method described by McCloy et al. (35) as following CTCF = Integrated Density − (area of selected image × mean background fluorescence). Unless otherwise indicated, the fold change in the CTCF when compare to the average data from the from healthy normal colonic tissue images was calculated.

### T Cell Isolation, Activation, and Polarization

Peripheral blood mononuclear cells were prepared from the blood of healthy donors using density gradient centrifugation over Ficoll-Plaque Plus (Amersham Bioscience, Piscataway, NJ, USA) according to the manufacturer’s instructions. Naive human CD4^+^ T cells were then purified from these peripheral blood mononuclear cells by negative selection using a commercially available Naive CD4^+^ T-cell isolation kit II (Miltenyi Biotec, Auburn, CA, USA) according to the manufacturer’s instructions. Negative selection was chosen to avoid accidental activation of CD4^+^ T cells during purification. Purified human naive CD4^+^ T cells were activated using Dynabeads™ Human T-Activator CD3/CD28 for T Cell Expansion and Activation kit (ThermoFisher Scientific, Grand Island, NY, USA) according to the manufacturer’s instructions. In some experiments CD45RA^+^ CD4^+^ T cells were polarized toward Th1 prior the use in a coculture experiments. For this purpose 1 × 10^6^ of CD45RA^+^ CD4^+^ T cell were cultured in standard MEM in presence of the T-Activator CD3/CD28 T Cell Expansion and Activation kit with a Th1 differentiation cocktail (rIL-12, 25 ng/ml + anti-hIL-4, 5 µg/ml). On day 4 (expansion step) cells were harvested, counted, and resuspended at the concentration 1 × 10^6^ cell/ml and cultured in MEM media supplemented with the IL-2 (20 U/ml) and Th1 differentiation cocktail up to day 7. On day 7 anti-CD3/CD28 beads were removed according to the T-Activator CD3/CD28 for T Cell Expansion and Activation kit instruction and described above procedure was repeated for total of 14 days. The purity of isolated CD45RA^+^ CD4^+^ T cells (>98%) and efficiency of the Th1 polarization was controlled by flow cytometry prior the use of cells in the CMF-T cell cocultures. In some experiments, T cells were labeled with Carboxyfluorescein Diacetate Succinimidyl Ester (CFSE) using the cell trace™ CFSE cell proliferation kit (Molecular Probes, Inc., Eugene, OR, USA) according to the manufacturer’s instructions.

### CMF:T Cell Coculture Experiments

Primary adherent CMFs were growth until 90% confluency in 24-well plates (7-day culture) in a standard MEM media, then used in the CMF:T cell coculture experiments. Activated or unprimed CD4^+^ T cells were cocultured in 24-well plates in the presence or absence of CMFs in ration 2.5:1, respectively. mAbs against the studied co-stimulatory molecules, PD-L1 Fc human fusion protein or isotype controls were added to the cocultures (when necessary) at a final concentration 1 µg/ml. Cocultures or control monocultures were then incubated for 5 days maximum at 37°C in 5% CO_2_.

### Primary CMFs Transfection With Small Interfering RNA (siRNA)

Small interfering RNA technology was used to knockdown expression of PD-Ls molecules in human primary CMF isolates as described previously (16). Breifly, negative siRNA controls are included in each experiment. Stealth siRNAs Set of three siRNA probes to the conservative domains of PD-L1, PD-L2, or negative siRNA control were purchased from Thermo Fisher Scientific. Transfection of CMFs was performed using Human Dermal Fibroblast Nucleofector kit according to the manufacturer instruction (Lonza, Allendale, NJ, USA). The efficiency of the downregulation of the PD-L1 or PD-L2 expression by specific siRNA set was controlled by real-time RT-PCR and flow cytometry.

### Real-Time RT-PCR

Real-time RT-PCR analysis was performed according to the two-step RT real-time PCR protocol as previously described (36). The appropriate assays-on-demand™ gene expression mix (ThermoFisher Scientific) for human the gene of interest and housekeeping gene (a 20× mix of unlabeled PCR primers and TaqMan^®^ MGB probe, FAM™ dye-labeled) and 2 µl of cDNA were added to the PCR reaction step. The reactions were carried out in a 20-µl final volume using a Bio-Rad Q5 real-time PCR machine according to the following protocol: 2 min at 50°C, 10 min at 95°C (1 cycle), and 15 s at 95°C and 1 min at 60°C (45 cycles).

### Flow Cytometry

Single- and multi-color immunostaining were performed according to standard eBioscience surface and intracellular FACS staining protocols. Cells were analyzed by flow cytometry using LSRII cytometers (BD Biosciences) per the manufacturer’s procedure. Area, height, and width parameters for forward and side scatters (FSC and SSC, respectively) were used to discriminate single live cells. An additional gate was set up based on the negativity for the fixable viability dye eFluor^®^ 780 (eBIoscience), which was added during the surface marker staining to exclude dead cells from the analysis. Flow cytometry data were analyzed using FACSDiva 6.3 (Becton Dickinson) software.

### Cytokine Production

For the analysis of cytokine production in the freshly obtained colonic specimens, the mucosa tissue was cut to ~8 mg pieces and incubated in complete RPMI for 12 h. Supernatants were collected and cytokines were measured by multiplex bead array (EMD Millipore, Bellerica, MA, USA) using a Luminex™200 according to the instructions from manufacture. Cytokine analysis was also performed on conditioned media from CMF:T cell cocultures and the cytokine profiles in coculture supernatants were analyzed as described above.

### Statistical Analysis

The results were expressed as the mean ± SE of data obtained from at least three independent experiments done with at least duplicate sets per experiment. Unless otherwise indicated, differences between means were evaluated by one way ANOVA in GraphPad Prism 5. Values of *P* < 0.05 were considered statistically significant.

## Results

### PD-L1 mRNA Level Is Increased in Colonic Mucosa From UC, but Decreased in CD Colitis

**Figure 1:**
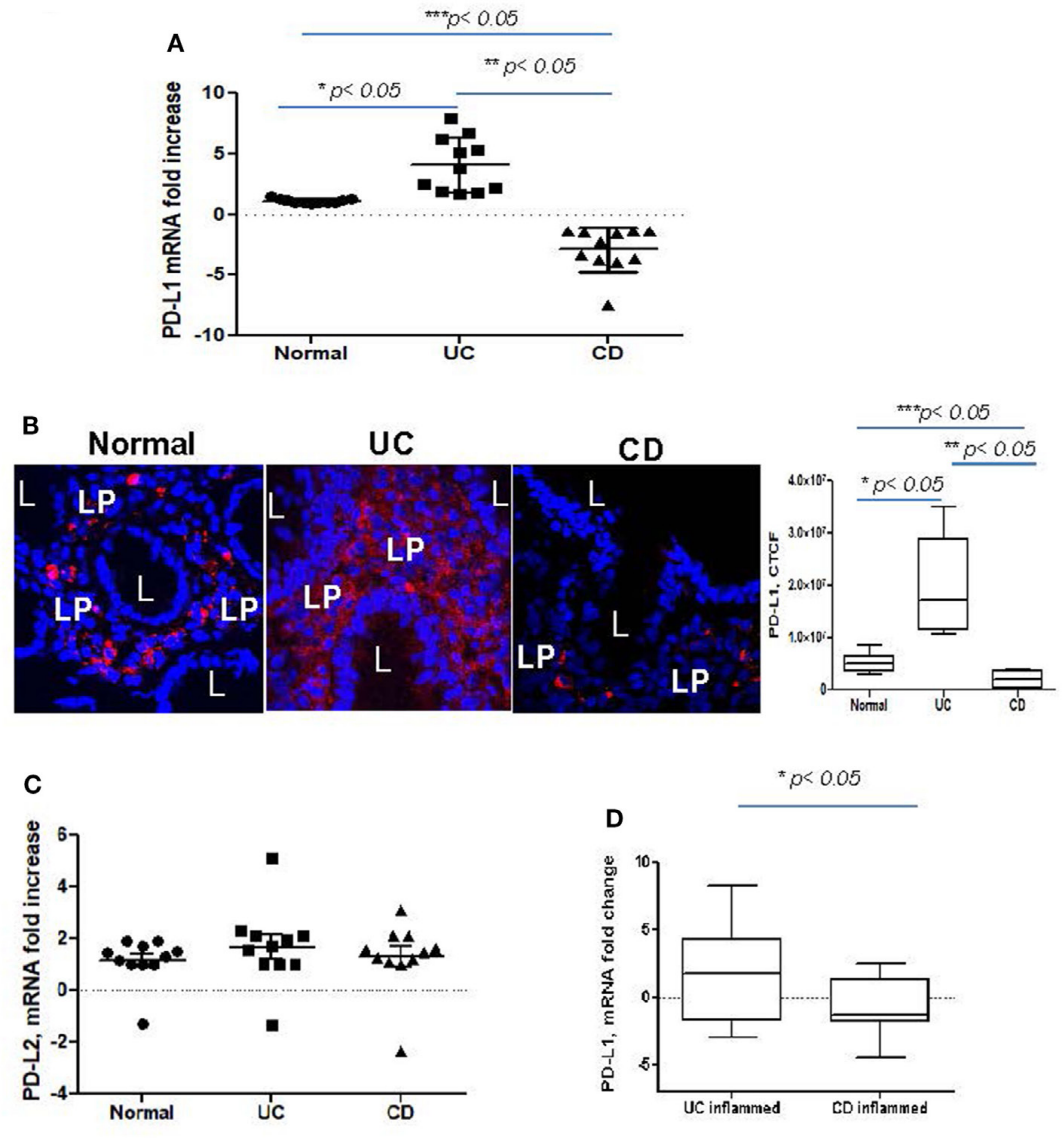
Programmed death-ligand 1 (PD-L1) is increased in ulcerative colitis (UC), but decreased in Crohn’s disease (CD) colonic mucosa. **(A)** PD-L1 mRNA levels in UC and CD colonic mucosa were compared to normal tissue controls obtained from healthy individuals (real-time RT-PCR analysis). The means ± SEM are shown as the results of duplicates of each tissue sample, *n* = 11 per group. **(B)** PD-L1 protein expression *in situ* was increased in UC and somewhat decreased in CD. In these experiments, frozen colonic tissue sections were stained with DAPI to identify cell nuclei (blue) and anti-PD-L1 monoclonal antibodies (clone MIH, in red) and analyzed by confocal microscopy (63×). A representative cross sections and summary of fold changes in the corrected total cell fluorescence (CTCF) of PD-L1 expression in UC, CD, and normal human colonic mucosa (the means ± SEM are shown, *n* = 10 per group) are shown, L, lumen; LP, lamina propria, *p < *0.05*. **(C)** PD-L2 mRNA levels in UC and CD colonic mucosa were compared to normal tissue controls obtained (real-time RT-PCR analysis). The means ± SEM shown are the results of duplicates of each tissue sample, *n* = 11 per group. **(D)** PD-L1 mRNA levels in UC and CD inflamed colonic mucosa was normalized to its matched, non involved tissue controls (real-time RT-PCR analysis). The means ± SEM shown are the results of duplicates of each tissue sample, *n* = 20 per group.

Despite recent evidence pointing to a critical role of PD-L1 and PD-L2 in the maintenance of gut tolerance, their expression within the two major subtypes of IBD remains contradictory. Therefore, we examined the level of PD-L1 and PD-L2 mRNA expression in colonic mucosa from adult patients with chronic UC and CD colitis. A significant increase in total PD-L1 mRNA expression in UC colonic mucosa was noted when compared to normal healthy controls. By contrast, the total tissue PD-L1 mRNA expression was decreased in CD (Figure 1A). A similar observation was made using *in situ* analysis: PD-L1 protein was mostly upregulated in both UC mucosal lamina propria (LP) and to a less extent in epithelium but decreased in CD LP compared to the healthy controls (Figure 1B). In contrast, no change in PD-L2 was observed in either IBD type when compared to normal controls (Figure 1C). Analysis of colonic tissue obtained from surgical resections demonstrated that PD-L1 expression was significantly upregulated in the inflamed mucosa from UC and downregulated in CD when compared to the non-inflamed mucosal samples from the same subject (i.e., matched samples) (Figure 1D). These observations suggest that changes in PD-L1, but not PD-L2 may be contributing to the immunopathogenesis of IBD.

### Expression of PD-L1 by CD90^+^ Mesenchymal Stromal Cells Depends on the Subtype of IBD: High in UC, but Low in CD

**Figure 2:**
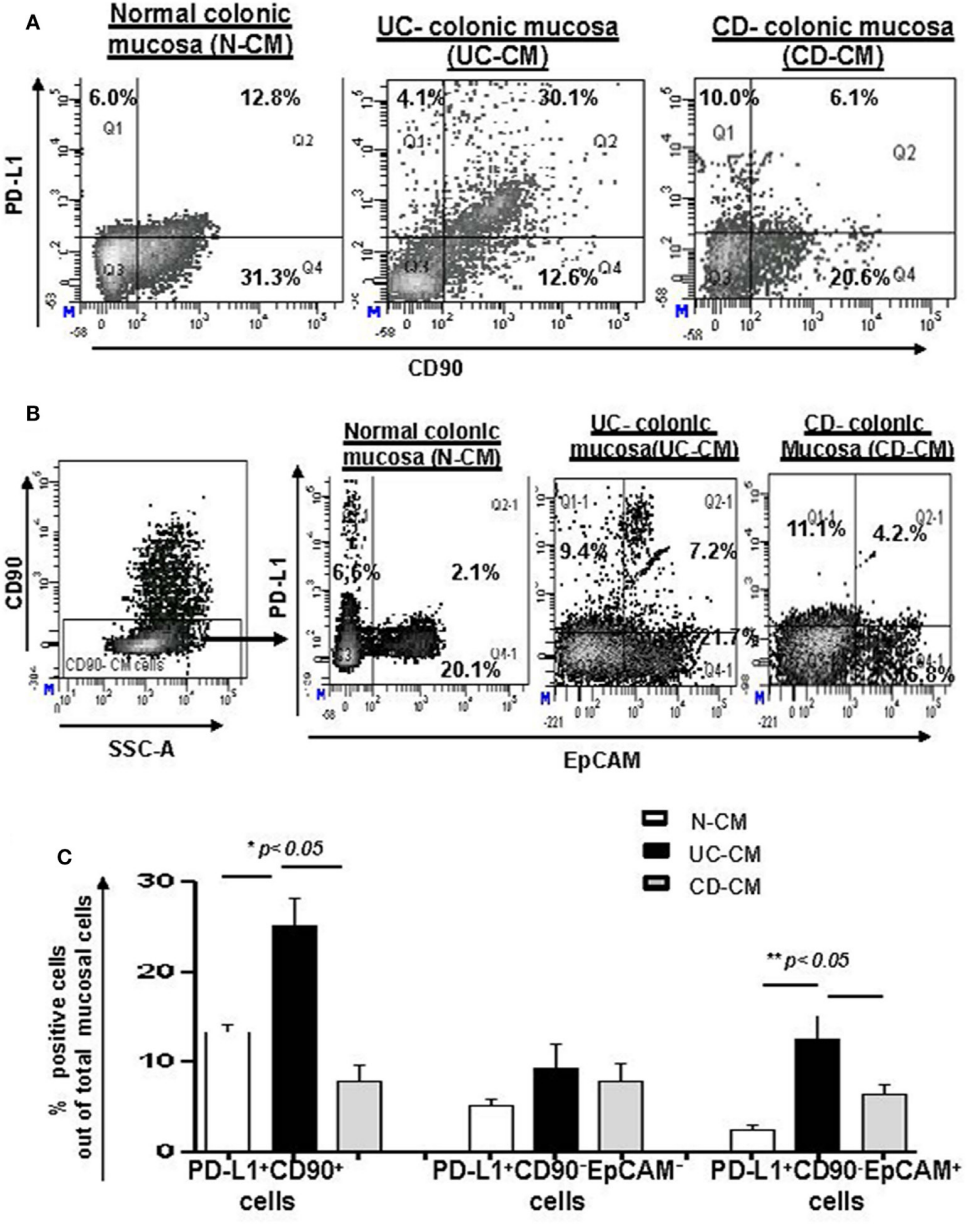
The number of programmed death-ligand 1 (PD-L1) expressing CD90^+^ colonic stromal cells (CMFs) is increased in ulcerative colitis (UC), but decreased in Crohn’s disease (CD) colitis. Freshly digested Normal (N), UC, and CD colonic mucosal single cell suspensions (CM) were prepared and immunostained and analyzed by multi-color flow cytometry. Live events were gated and mucosal cells (CMs) were analyzed for the PD-L1 expression. **(A)** A representative density plot for the flow cytometry analysis for the expression of PD-L1 by CD90^+^ CMFs. **(B)** A representative density plot for the flow cytometry analysis for the expression of PD-L1 by EPCAM^+^ cells out of CD90^−^ colonic mucosal (CD90^−^ CM) cells. **(C)** Summary of the PD-L1 expression by CD90^+^ EpCAM^−^ cells (CMFs) CD90^−^ EpCAM^+^ cells (epithelial cells) and CD90^−^EpCAM^−^ professional immune cells in normal, UC-CM, and CD-CM preparations (flow cytometry analysis). Values are expressed as a mean of percentage positive cells ± SD, *n* = 9 per group.

**Figure 3:**
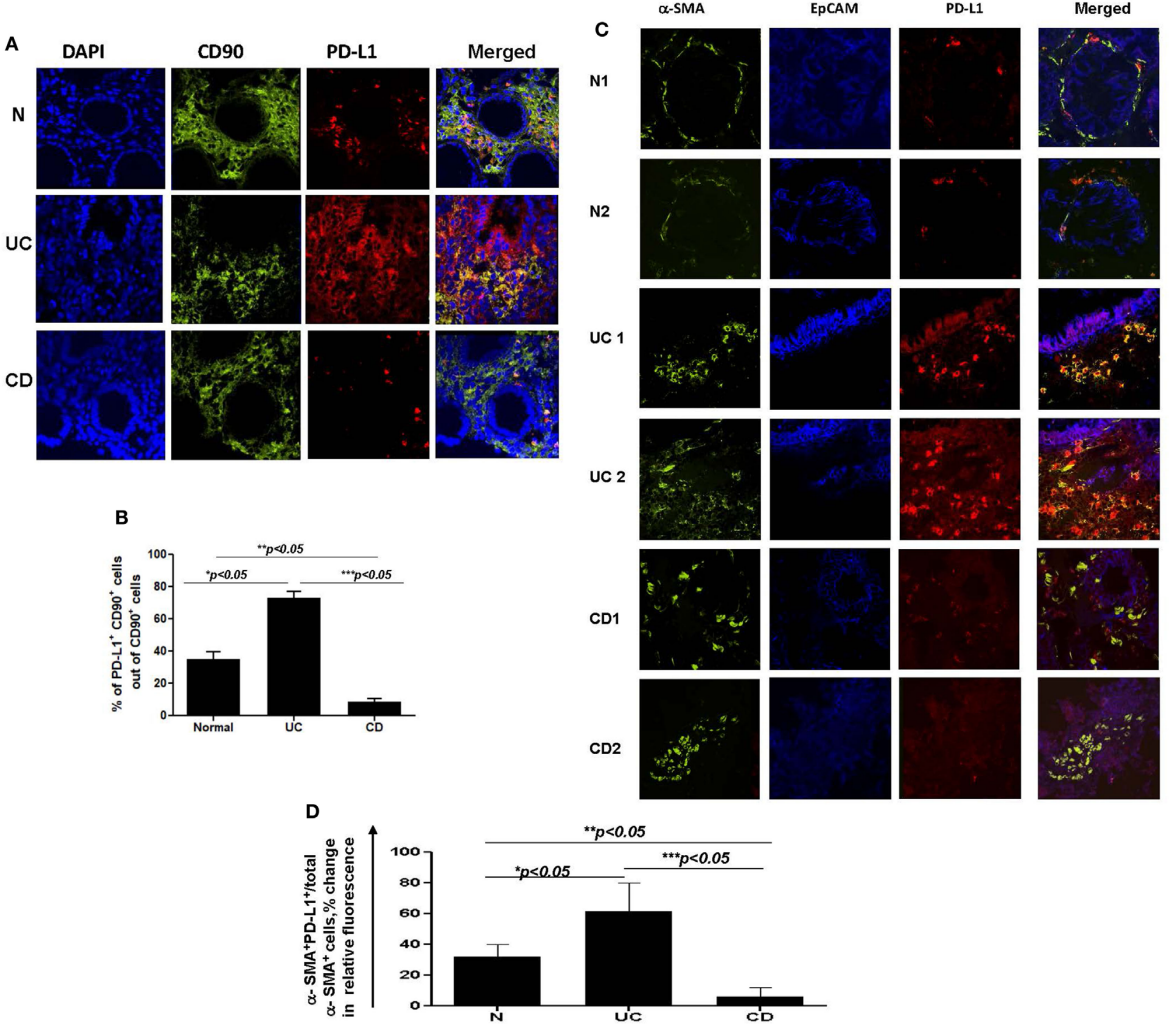
**(A)** The number of programmed death-ligand 1 (PD-L1) expressing CD90^+^ mesenchymal cells is increased in ulcerative colitis (UC) and decreased in Crohn’s disease (CD) colonic mucosa. Confocal microscopy analysis of a representative cross sections of UC, CD, and normal human colonic mucosa (*n* = 10 per group) were performed. Cell nuclei (in blue) were stained with DAPI. CMFs may be identified by morphology, *lamina propria* location, and staining with anti-human CD90 monoclonal antibodies (mAbs) conjugated to AF^®^488 (clone 5E10, in green). PD-L1 staining (in red) was performed using AF^®^633-labeled anti-PD-L1 human mAbs (clone MIH1). Co-localization of PD-L1 within CD90^+^ CMFs shown in yellow-orange (arrow) on merged images. **(B)** Summary of changes in the PD-L1 expression by CD90^+^ CMFs *in situ*. Image J software was used for the quantification analysis as described in Section “Materials and Methods.” Data were expressed as means ± SEM of percentage of the changes in the count of corrected total cell fluorescence (CTCF) of PD-L1 positive CD90^+^ cells (yellow-orange color formation) over total CD90^+^ CMFs (both, green and yellow-orange stained cells), *n* = 10 per group, *p < *0.05*. **(C)** Changes in PD-L1 expression in mesenchymal *stromal cells* of inflamed inflammatory bowel disease colonic mucosa are mainly associated with α-smooth muscle actin (α-SMA^+^) myofibroblasts. Confocal microscopy images of two representative cross sections from UC, CD, and normal human colonic mucosa (*n* = 7 per group) are shown. Activated fibroblasts(myofibroblasts), were detected by anti-α-SMA mAb (green), and epithelial cells were identified with anti-EpCAM mAb (in blue). Co-localization of PD-L1 with epithelial cells and α-SMA^+^ myofibroblasts results in formation of magenta and yellow-orange color, respectively, on merged images. **(D)** Summary of the changes in the PD-L1 expression by α-SMA^+^ cells (a.k.a. myofibroblasts) *in situ*. Image J software was used for the quantification analysis. Data were expressed as means ± SEM of percentage of the changes in the count of CTCF of PD-L1 positive α-SMA^+^ cells (yellow-orange color formation) over total α-SMA^+^ myofibroblasts (both, green and yellow-orange stained cells), *n* = 7 per group, *p < 0.05.

CD90^+^ stromal (myo)fibroblasts (CMFs) are abundant in the normal and IBD mucosal LP and can be identified in humans (but not mice) by surface expression of the mesenchymal lineage marker CD90 (33, 37). Previously, we reported that CMFs are the major cell type expressing PD-L1 in colonic LP under homeostasis (16). Thus, to examine cellular compartmentalization of PD-L1 expression in inflamed IBD tissue, we performed immunostaining followed by flow cytometry analysis of fresh single cell suspension preparations of whole layer of human colonic mucosa (epithelium and LP). We observed a significant increase in PD-L1^+^CD90^+^ CMFs in UC. In contrast, a decrease in PD-L1 expressing CMFs was noted in the CD colonic mucosa (Figures 2A,C). Interestingly, while not reaching significance, an overall relative loss of CMFs was occasionally observed in CD mucosa (Figure 2A). The last one may be due to degeneration of the pericryptal CMFs, which was previously observed by us in IBD tissue (38). Analysis of IBD colonic tissue *in situ* confirmed the flow cytometry observations that the PD-L1 expressing CD90^+^ cell population is strongly increased in the inflamed UC colonic mucosa (Figure 3, formation of yellow-orange color on merged images). In contrast, a decrease in PD-L1 expression In the CD90^+^ cell population was seen in CD (Figures 3A,B). We observed a moderate increase in PD-L1^+^EpCAM^+^ epithelial cells in UC. Epithelial PD-L1 was increased, but to a lesser extent in CD than that the increase seen in UC, suggesting that a redistribution of PD-L1 expression (decrease in LP cells and slight increase in epithelial cells) may occur in CD (Figures 2B,C). The flow cytometry observations were confirmed by tissue immunostaining *in situ* followed by confocal microscopy analysis (Figure 3C, formation of magenta color on merged images). Additionally, some increase in PD-L1 expressing CD90^−^ EpCAM^−^ CMs were observed in both forms of IBD (Figure 2C).

Taken together, our data suggest that epithelial cells and stromal cells (CMFs) respond to the IBD chronic inflammation with changes in PD-L1 expression. Further, colonic LP PD-L1 is differentially expressed in UC (increased) vs CD (decreased). Finally, CD90^+^ mesenchymal cells (CMFs) are the cell phenotype associated with differential expression of PD-L1 in the two major forms of IBD.

### Changes in PD-L1 Expression in IBD-Derived Colonic Mesenchymal Stromal Cells Are Mostly Associated With α-SMA^+^ Myofibroblasts

To further examine the CD90^+^ population expressing PD-L1, staining was undertaken using the myofibroblast marker, α-SMA. CD90 is a pan-mesenchymal lineage marker in the human identifying both mesenchymal stem cells (MSCs), fibroblasts, and myofibroblasts (37, 39). Recent publications provide strong evidence for a slow cycling, α-SMA negative tissue MSC population in the LP of intestinal mucosa (40). In contrast, α-SMA marks mostly differentiated LP mesenchymal cells otherwise known as myofibroblasts. Thus, next, we investigated PD-L1 expression by α-SMA^+^ CMFs in IBD inflamed colonic mucosa compared to the normal mucosa obtained from normal healthy controls. Co-immunostaining of UC tissue for α-SMA (in green) and PD-L1 (in red) demonstrated a strong increase in PD-L1 expression mostly co-localized with α-SMA^+^ myofibroblasts (Figures 3C,D). Analysis of α-SMA^+^ CMFs in CD colitic mucosa demonstrated that PD-L1 expression was either decreased in comparison to the normal tissue or completely lost (Figures 3C,D).

### Changes in PD-L1 Expression Modify IBD-CMF-Mediated Suppression of CD4^+^ T Cell Proliferation

**Figure 4:**
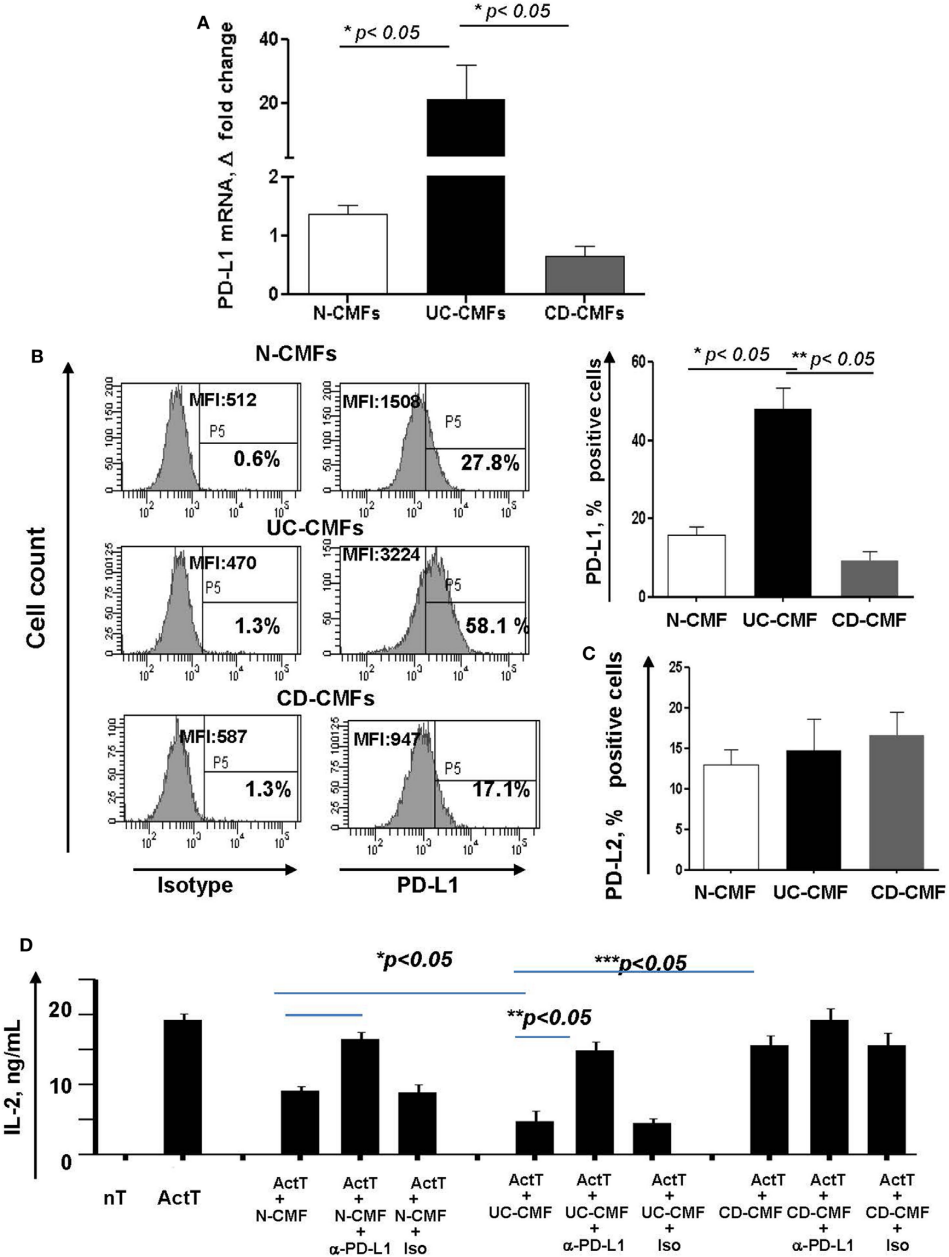
An increase in surface programmed death-ligand 1 (PD-L1) by UC-CMFs and a decrease by CD-CMFs modify cell-mediated suppression of IL-2 production byCD3-/CD28-activated CD4^+^ T cell IL-2 production. **(A)** PD-L1 mRNA (real-time RT-PCR analysis) and **(B)** surface protein (flow cytometry analysis) expression was increased in 6–7 days, 90–100% confluent primary cultures of UC-CMFs, but decreased in CD-CMFs when compared to N-CMFs. Representative flow cytometry histogram and summary of the surface PD-L1 expression shown on panel **(B)**. The means ± SEM are shown, *n* = 8 per group. **(C)** PD-L2 surface expression on primary UC-, CD-, and N-CMFs (flow cytometry analysis); the means ± SD are shown, *n* = 8 per group. **(D)** CMFs were cocultured with allogeneic CD3-/CD28-preactivated naive CD4^+^ T cells (Act T cells) at a ratio of 1:2.5 for 72 h in 24-well plates. PD-L1 blocking mAbs (clone MIH1) or isotype controls were added in the concentration 1 µg/ml. IL-2 production was analyzed using singleplex cytokine analysis. The means ± SD are shown, *n* = 5 allogeneic donor pairs per group, two experimental replicates each.

(Myo)fibroblasts isolated from normal (N-) and cancer human mucosal GI tissues maintain their phenotype and activity in culture (16, 41, 42). Thus, to assess the functional significance of the changes in PD-L1 expression by colonic MFs (CMFs) in IBD, we isolated CMFs from CD and UC colonic mucosa and compared them to N-CMFs. Both UC- and CD-CMF isolates maintained their differential high or low profile of PD-L1 expression in culture as determined by mRNA and surface protein analysis (Figures 4A,B). In contrast, no significant changes were noted in surface PD-L2 expression on these cells (Figure 4C).

Previously, we reported that N-CMFs-suppress activated T effector cell proliferation and IL-2 (a cytokine produced by actively proliferating T cells) *via* a PD-L1- and PD-L2-mediated mechanism (16). Thus, we next examined how changes in PD-L1 expression modify the capacity of IBD-CMFs to suppress activated CD4^+^ T cell proliferation. In these assays, CD45RA^+^CD4^+^ T cells were isolated from peripheral blood mononuclear cells of healthy volunteers, activated with anti-CD3/anti-CD28 mAbs and then incubated in the presence of UC-, CD-, or N-CMFs. We observed that UC-CMFs exhibited stronger suppression of IL-2 production when compared to N-CMFs, although this suppression did not reach statistical significance. In contrast, CD-CMFs exhibit significantly less suppression of T cell proliferative response (Figure 4D and Figure S1 in Supplementary Material). The N- and UC-CMF-mediated suppressive capacity was PD-L1 dependent since addition of PD-L1 blocking antibodies, but not isotype control, reversed suppression of IL-2 production in these cocultures (Figure 4D). While the mechanisms implicated in the differential impairment of PD-L1 expression by IBD-derived CMFs are currently unknown, these data suggest that UC-derived CMFs suppress activated T cell proliferation as we previously observed for N-CMFs (16). In contrast, CD-CMF-mediated suppression is significantly decreased, possibly due to the combination of the decreased expression of PD-L1 in conjunction with other not yet well characterized factors that may enhance IL-2 production by T cells.

### Changes in PD-L1 Expression Modify IBD-CMF-Mediated Regulation of Tbet and IFN-γ Production by CD3/CD28-Activated and Differentiated Th1 CD4^+^ T Cells

**Figure 5:**
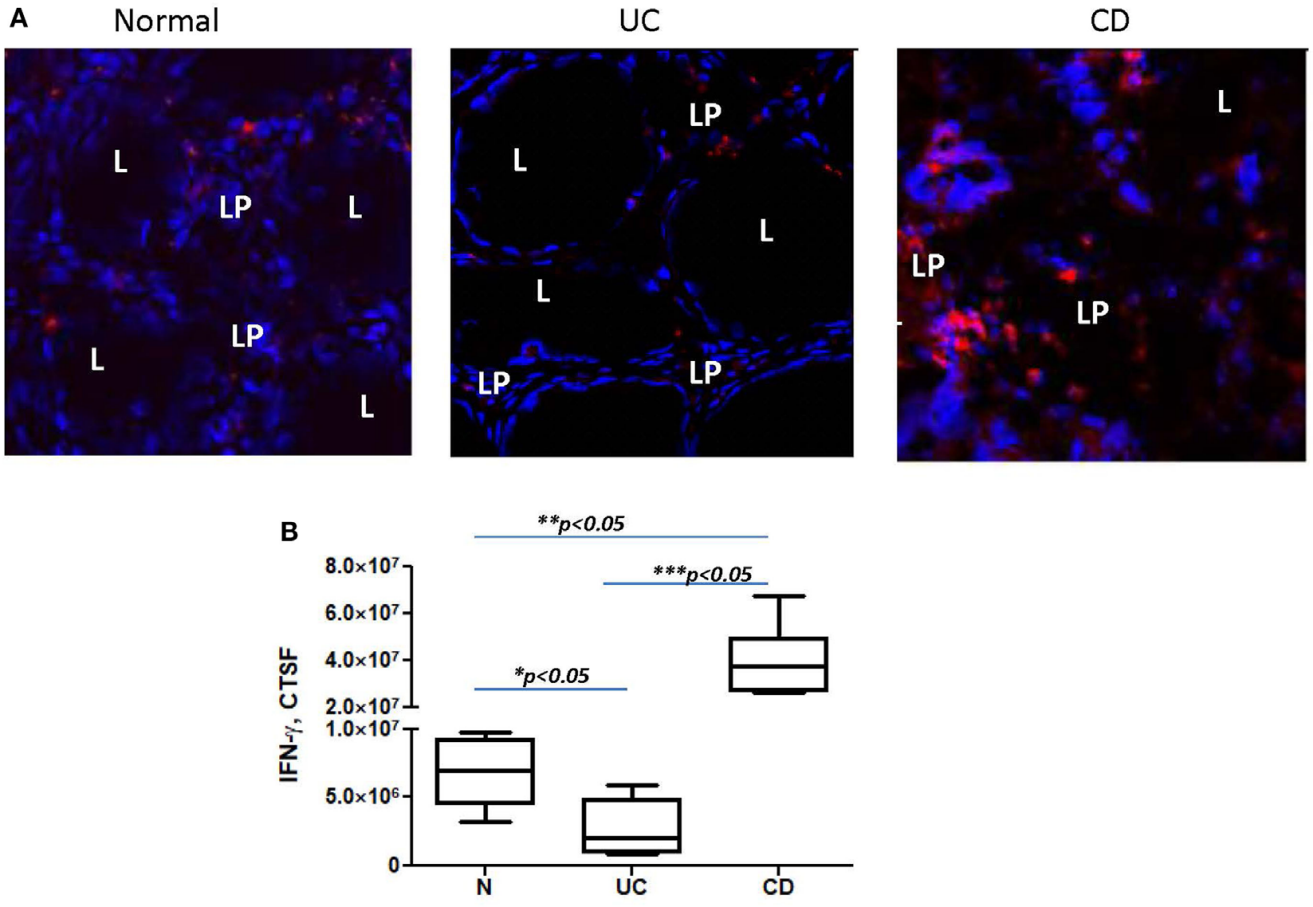
IFN-γ expression is decreased in the colonic mucosa of ulcerative colitis (UC) but increased in Crohn’s disease (CD) when compared to normal controls. In these experiments, frozen colonic tissue sections were stained with DAPI to identify cell nuclei (blue) and anti-IFN-γ monoclonal antibodies (clone 4S.B3, in red) and analyzed by confocal microscopy, objective used is 63×. **(A)** Representative cross sections of UC, CD, and normal human colonic mucosa and **(B)** summary of fold changes in the corrected total cell fluorescence (CTCF) of PD-L1 expression in UC, CD, and normal human colonic mucosa (the means ± SEM are shown, *n* = 5 per group), L, lumen; LP, lamina propria.

**Figure 6:**
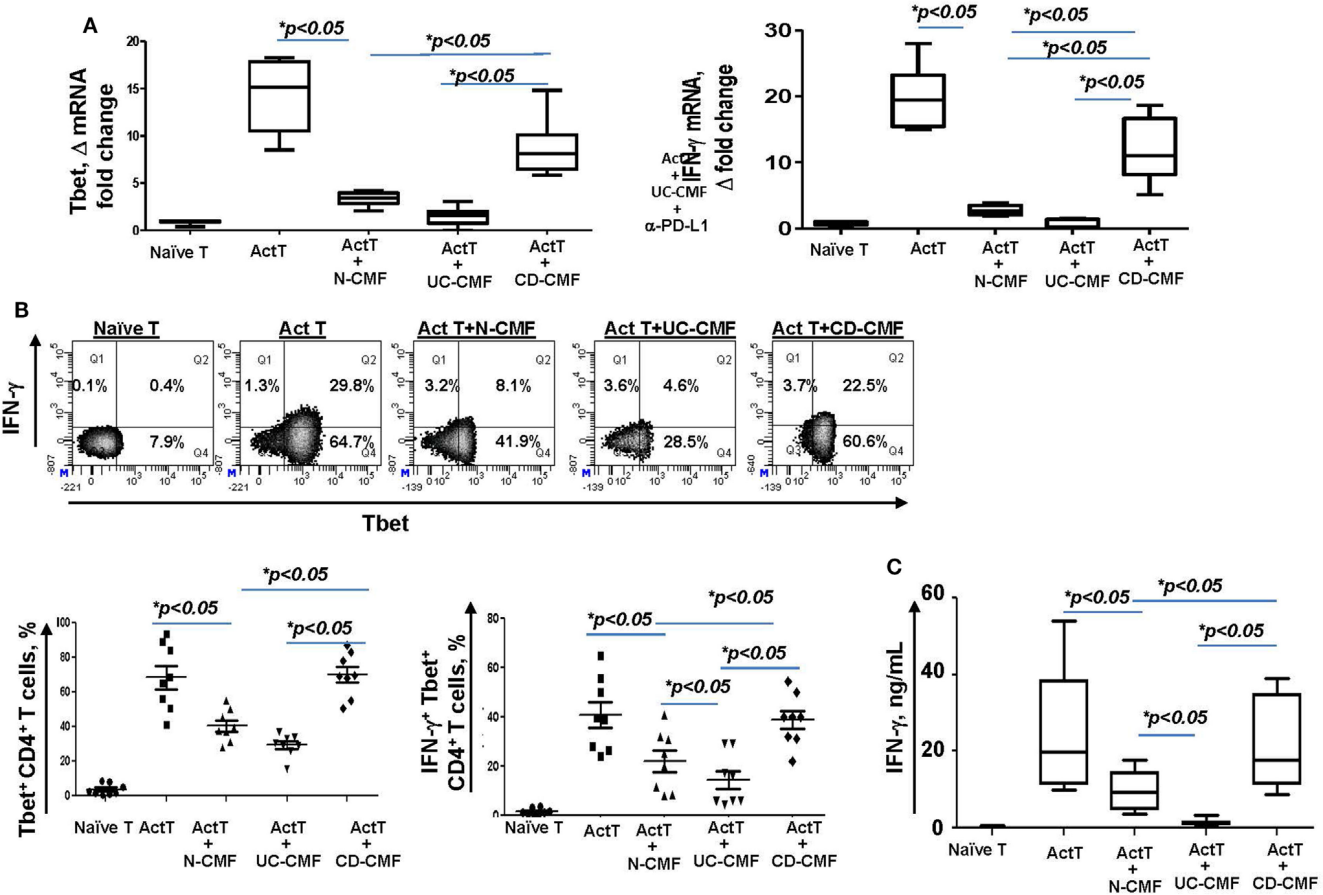
UC-CMFs demonstrate strong suppression of Th1 type responses in the CD3/CD28-activated CD4^+^ T cells, while CD-CMF-mediated suppression was significantly reduced. **(A)** Tbet and IFN-γ mRNA expression by the T cells was analyzed using real-time RT-PCR analysis. The means ± SEM are shown, *n* = 4 allogeneic donor pairs per group, two experimental replicates each. **(B)** The percentage of the T cells expressing Th1 transcription factor Tbet and the Th1 cytokine IFN-γ was analyzed using intracellular immunostaining for flow cytometry. The means ± SED are shown, *n* = 8 allogeneic donor pairs per group. **(C)** IFN-γ production was analyzed using singleplex cytokine analysis. The means ± SD are shown, *n* = 8 allogeneic donor pairs per group, two experimental replicates each.

Programmed death-ligand 1 is critical to the regulation of the balance between Th1 and Th2 type immune responses *in vivo* (11). Previously, we have shown that N-CMFs suppress IFN-γ production by activated T cells in a PD-L1-dependent manner (32). An increase in the IFN-γ-producing Th1 cells in CD LP and a decrease in UC LP have been recently observed by several investigators (43–45). We also observed an increase in IFN-γ levels in the inflamed mucosa of patients with CD colitis (Figure 5). Next, we determined how changes in PD-L1 expression within IBD-CMFs modify their capacity to regulate Th1/Th2 type responses in activated CD4^+^ T cells. Priming of activated T cells with UC-CMFs led to stronger suppression of Tbet mRNA expression (Th1 response) in the T cells than did priming with N-CMF. Similar data were observed for IFN-γ expression (Figure 6A). In contrast, priming of activated T cells with CD-CMFs failed to suppress Th1 type responses (Figure 6A). These data were confirmed on the protein level by flow cytometry: UC-CMFs demonstrated stronger suppression of the IFN-γ^+^Tbet^+^ cells when compared to N-CMFs, while CD-CMF-mediated suppression of the IFN-γ^+^Tbet^+^ was significantly reduced or lost (Figure 6B). These findings were confirmed by measuring IFN-γ secretion by bead array (Figure 6C).

### PD-L1, but not PD-L2, Is Critically Involved in the Alteration of CMFs-Mediated Suppression of Th1 Type Responses in IBD

**Figure 7:**
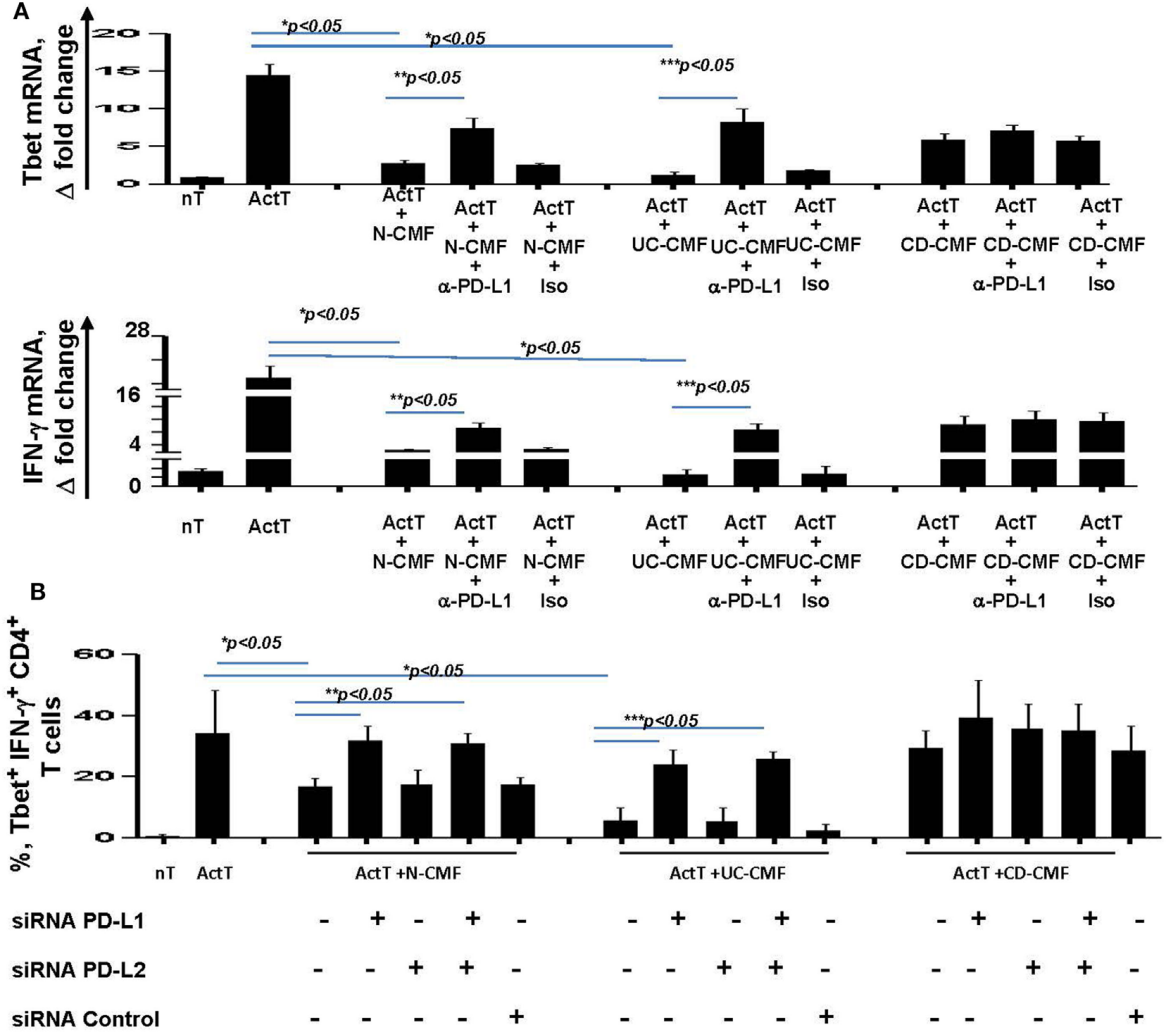
CMF-mediated suppression of Th1 type responses depend on the level of expression of the programmed death-ligand 1 (PD-L1), but not PD-L2. **(A)** N-, UC-, and CD-CMFs were cocultured with allogeneic CD3/CD28-activated naive CD4^+^ T cells in presence/absence of PD-L1 blocking monoclonal antibodies (clone MIHI) or isotype control. Tbet and IFN-γ mRNA expression by the T cells was analyzed using real-time RT-PCR analysis. The means ± SEM are shown, *n* = 4 allogeneic donor pairs per group, two experimental replicates each. **(B)** N-, UC-, and CD-CMFs transfected or not with small interfering RNA (siRNA) specific to PD-L1, PD-L2, or siRNA control were cocultured with allogeneic CD3/CD28-preactivated naive CD4^+^ T cells. The percentage of the T cells expressing Tbet and IFN-γ was analyzed using intracellular immunostaining followed by flow cytometry. The means ± SD are shown, *n* = 4 allogeneic donor pairs per group, two experimental replicates each.

**Figure 8:**
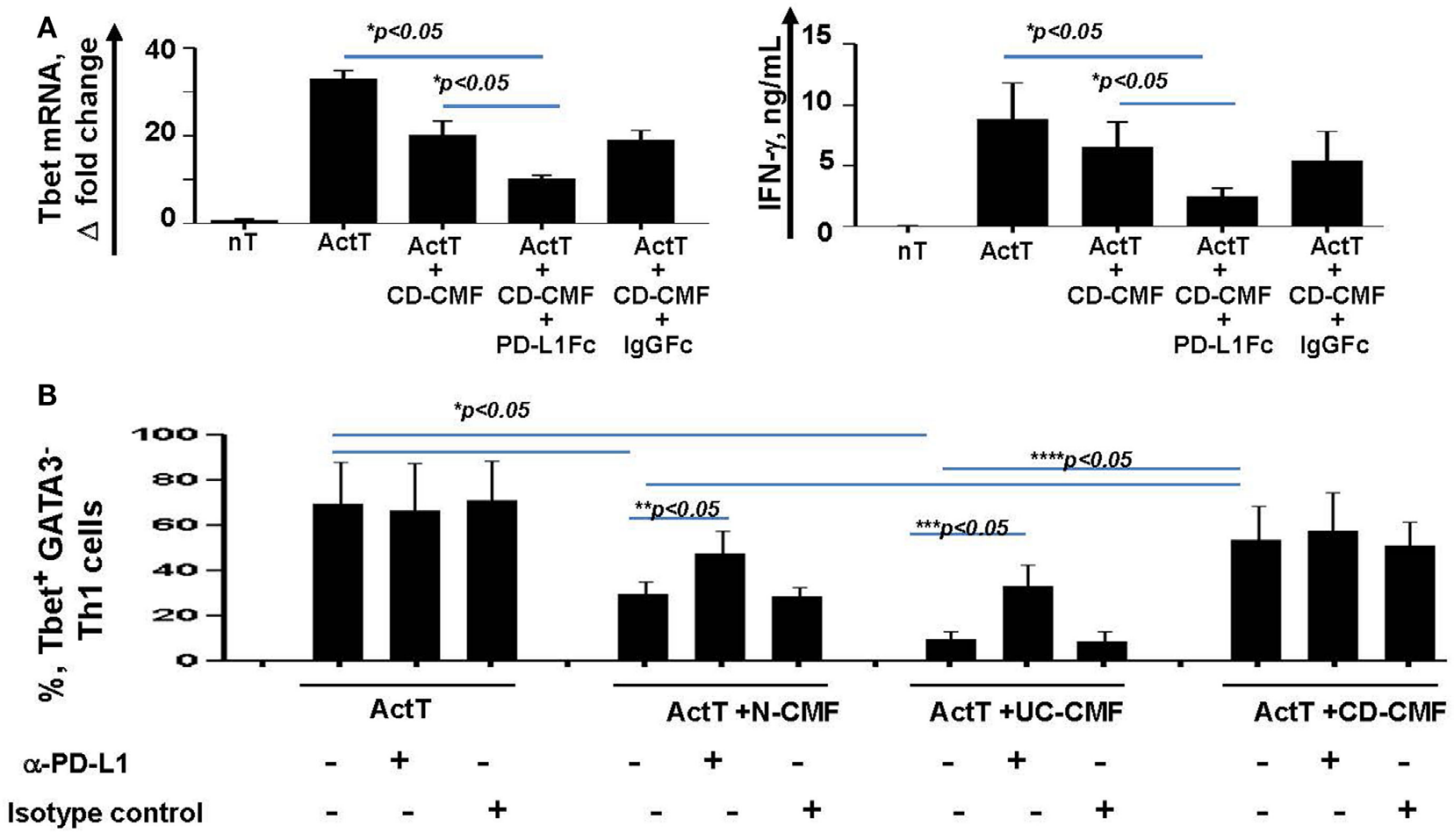
**(A)** CD-CMFs were cocultured with allogeneic CD3/CD28-preactivated naive CD4^+^ T cells in presence/absence of the PD-L1Fc or IgG Fc control. IFN-γ expression (real-time RT-PCR) and secretion (singleplex cytokine analysis) were analyzed. The means ± SEM are shown, *n* = 3 allogeneic donor pairs per group, two experimental replicates each. **(B)** Th1 cells were differentiated from CD4^+^ naïve T cells and cocultured with N-, UC-, and CD-CMFs in presence/absence of the PD-L1 blocking monoclonal antibodies or isotype control. Percentage of the T cells expressing the Th1 transcription factor Tbet was analyzed by flow cytometry. The means ± SED are shown, *n* = 7 allogeneic donor pairs per group.

Blocking PD-L1 with specific antibodies significantly reduced the ability of N- and UC-CMFs to suppress both Tbet and IFN-γ mRNA expression by cocultured T cells (Figure 7A). Since activated T cells are capable of expressing low levels of PD-L1, we then confirmed the primary role of CMFs using PD-L1 specific siRNA (Figure 7B). A contribution of PD-L2 to the suppression of the Th1 type responses was previously observed in the animal models of airway hyperreactivity and in infectious diseases (46, 47). Because both N- and IBD-derived CMFs expressed basal levels of PD-L2 and PD-L1, to determine the relative contribution of either ligand to the CMF-mediated suppression of Th1 immune response, experiments were included studies in which PD-L2 was silenced using specific siRNA. Since silencing of PD-L2 in CMFs did not significantly change the CMF-mediated suppression of the IFN-γ^+^Tbet^+^ cells (Figure 7B), we concluded that CMF-mediated suppression of the Th1 type responses was specific to PD-L1, but not PD-L2. We next determined if compensation of this lack of the immune checkpoint molecule by using PD-L1 hIgG1 fusion protein (PD-L1Fc) which may bind to the Fc receptor on CD-CMFs, compensates to these cells decreased PD-L1 expression, and might restore the ability of CD-CMFs to suppress activated CD4^+^ T cells since CD-CMFs express low levels of PD-L1. Indeed, addition of the PD-L1 Fc, but not IgG Fc (as a control), to the CD-CMFs: activated T cell cocultures resulted in partial restoration of CD-CMF-mediated suppression of the Tbet mRNA expression and IFN-γ production by cocultured T cells (Figure 8A).

Finally, the effect of CMFs on T cells polarized toward a Th1 phenotype was examined. In these experiments, Th2 transcriptional factor GATA2 was used to assess the efficiency of the naïve CD4^+^ T cells polarization toward Th1. When compared to N-CMFs, UC-CMFs strongly suppressed the number of IFN-γ^+^Tbet^+^ Th1 cells. In contrast, CD-CMFs did not suppress Th1 cells (Figure 8B). The N- and UC- CMFs-mediated suppression of Tbet expression in Th1 cells was partially reversed when anti-PD-L1 blocking mAbs were added to the cocultures (Figure 8B).

Taken together, these data suggest that increased PD-L1 expression by CMFs in UC very likely contributes to the overt suppression of a Th1 type response. In contrast, downregulation of PD-L1 in CD-CMFs is likely to be an important contributor to the pathological increase in the inflammatory Th1 type responses observed in CD colonic mucosa.

## Discussion

Dysregulation of Th cell immune responses is a hallmark of IBD immunopathogenesis (2–4). However, the mechanisms involved in this dysregulation are far from elucidated. Recent studies in other diseases suggest that aberrations in PD-L1 and/or PD-L2 expression contribute to alterations in Th immune responses (46–49). However, very little is known about the role of these molecules in IBD. Data presented here suggest that aberrations in PD-L1 expression by colonic CMs may play a critical role in the imbalance in Th1/Th2 responses, and may contribute to the persistence of the chronic inflammation in IBD colitis. In other tissues and diseases, such as murine *Fasciola hepatica* infection and asthma, PD-L2 has been reported to negatively regulate Th1-mediated immunopathology (46) and promote Th2 type response (48), respectively. While we previously reported that PD-L2 contributes to the N-CMF-mediated suppression of T cell proliferation (16), here we show that PD-L2 is not critical to the CMF-mediated suppression of the Th1 type responses. An additional novel observation here is that, in contrast to a moderate upregulation of PD-L1 in both CD and UC lamina epithelium and other non mesenchymal cells, only CD90^+^ LP cells showed differential change in the expression of PD-L1 between these two subtypes of IBD. This observation suggests that changes in the immune regulatory function of CD90^+^CMFs may be functionally specific to the IBD subtype.

While several publications have reported an increase in PD-L1 expressing cells in IBD tissue (21, 23, 24), we have shown here that both PD-L1 mRNA and protein are differentially changed in IBD colitis: highly upregulated in UC and downregulated in CD. In contrast to other chronic diseases linked to the disruption of Th1/Th2 balance (46, 48), we did not observe significant changes in PD-L2 expression or its distribution within IBD colonic mucosa. PD-L1 has been shown also by others to suppress IFN-γ production by Th1 cells and promote Th2 immune responses (13, 49), and our studies concur in proposing in PD-L1 expression may be involved in the dysregulation of the Th helper responses observed in IBD.

In contrast to the study by Kanai et al. (21), we did not observe significant upregulation of PD-L1 in CD tissue, perhaps because we studied only active inflamed Crohn’s colitis, while this group examined tissues from CD stenosing ileitis (21). There is discordance as to the predominant cells expressing PD-L1 in IBD: macrophages and dendritic cell expression was noted in inflamed UC and CD colon (21) but in the IBD epithelium by others (24). However, those studies where performed mostly by flow cytometry, where selective enrichment and gating of the professional immune cells did not include CMF analysis. Furthermore, a deficiency in professional antigen-presenting cells PD-L1 induction was reported in small intestinal lymphoid patches in CD (25, 26). We confirmed that PD-L1 was moderately increased in the UC and CD epithelial cells as has been previously seen by Nakazawa et al (24). However, we demonstrated herein that a robust increase in PD-L1 expression in UC active inflamed mucosa is highly associated with cells bearing the mesenchymal phenotype, while a decreased PD-L1 expression is noted in these cells in CD.

Mesenchymal stem cells are believed to be major progenitors of CMFs (37, 40) and share the CD90 marker with CMF. MSC express PD-L1 (50, 51) and a recent study suggested that there may be tissue-resident MSC in the intestinal mucosa (40). Therefore, the possibility that some fraction of PD-L1^+^ cells may be MSCs cannot be excluded. However, staining for α-SMA, a marker of differentiated MSC, demonstrated that, at least in UC, the majority of PD-L1 expressing cells were identified as myofibroblasts. Further, the reduction of PD-L1 in CD may serve as a scientific rationale for the use of “PD-L1 competent” MSCs for the treatment of perianal CD where the major objective is to deposit these stem cells locally in fistulizing tracts to suppress the local inflammatory responses (52).

In addition to intestinal fibroblasts and myofibroblasts, PD-L1 expression also has been reported on other tissue fibroblast-like mesenchymal stromal cells, including hepatic stellate cells and decidual stromal cells (53). However, the role of the stromal cell-mediated PD-L1 signaling during chronic inflammatory diseases in those tissues is unclear. Herein, we demonstrated that the increase in PD-L1 expression by UC-CMFs and the decrease on CD-CMFs resulted in the respective increase and decrease of the CMF-mediated capacity to suppress Th1 type responses in both recently activated and fully differentiated Th1 cells demonstrating the importance of CMFs in the regulation of Th cell responses in these diseases. A disruption of Treg function has been observed in the IBD mucosa. We and other previously observed that PD-L1/PD-1 signaling also contributes to the induction of Treg (18, 54). In our previous study, we showed that while N-CMF induce generation of the suppressive Tregs, IBD-CMFs lose their capacity to generate fully suppressive Tregs (31). However, PD-L1-mediated signaling had only limited contribution to CMFs capacity to induce Treg (31). Further studies are needed to understand contribution of IBD-CMFs and its immune checkpoint repertoire to the dysregulation of the other type of the Th cells (e.g., Th17 cells) that contribute to the immunopathogenesis of the IBD.

Previously, we reported that basal CMF levels of PD-1 ligands are critical to the colonic mucosal tolerance (16, 32). Herein, we present evidence that increased PD-L1 expression suppresses Th1 cell activity in UC. In contrast, loss of CMF PD-L1 expression in the CD mucosal LP may contribute to the persistence of the Th1 inflammatory milieu in CD. Taken together, our findings suggest that while signaling through both PD-1 ligands is critical to the maintenance of the homeostatic tolerance, changes in the PD-L1 expression by IBD-CMFs may be among the critical factors supporting dysregulation of the Th1 type responses in both CD and UC. While further studies are required to understand the mechanism of these differential changes in the expression of PD-L1 in IBD-derived CMFs, and the mechanism of PD-L1’s effect on Th cell type, our data strongly support the significance of overall PD-L1 and especially CMF-mediated signaling in the immunopathogenesis of IBD. Further, while targeting PD-1 and its ligands has emerged as a successful therapeutic modality for the treatment of melanoma, renal, and lung cancers (55–57), one of the major irAEs of immune checkpoint therapy is development of diarrhea and colitis (58). The mechanism of this colitis remains to be clarified. Our data support the idea that PD-L1 expression may be a candidate for the development of novel diagnostic tissue biomarkers to differentiate CD and UC and this molecule could be considered a therapeutic target for IBD.

## Ethics Statement

This study was carried out in accordance with the recommendations of the Institutional Review Board at UTMB and UNM Health Sciences Center.

## Author Contributions

EB: study concept and design; acquisition of data; analysis and interpretation of data; and manuscript preparation and revision for important intellectual content. CG: acquisition of data; analysis and interpretation of data; and manuscript preparation and revision for important intellectual content. AS: acquisition of data and analysis and interpretation of data. JA: analysis and interpretation of data and manuscript preparation. MT: analysis and interpretation of clinical data and clinical material support. SQ: analysis and interpretation of data and clinical material support. RM: clinical material support; critical revision of the manuscript for important intellectual content. VS: analysis and interpretation of clinical data and clinical material support. TM: clinical material support; critical revision of the manuscript for important intellectual content. GR: clinical material support and critical revision of the manuscript for important intellectual content. VR: critical revision of the manuscript for important intellectual content and obtained funding. DP: study concept and design; analysis and interpretation of data; critical revision of the manuscript for important intellectual content; and obtained funding. IP: study concept and design; acquisition of data; study supervision; analysis and interpretation of data; manuscript preparation and revision for important intellectual content; and obtained funding.

## Conflict of Interest Statement

The authors declare that the research was conducted in the absence of any commercial or financial relationships that could be construed as a potential conflict of interest.

## Abbreviations

α-SMA, α-smooth muscle actin; CD, Crohn’s disease; CMs, mucosal cells; CMFs, CD90^+^ myofibroblasts/fibroblasts; IBD, inflammatory bowel disease; UC, ulcerative colitis; PD-L1, programmed death-ligand 1; PD-1, programmed cell death protein 1.
