# Supplementary material for: Expression of Programmed Death-Ligand 1 by Human Colonic CD90+ Stromal Cells Differs Between Ulcerative Colitis and Crohn’s Disease and Determines Their Capacity to Suppress Th1 Cells
Source: Front Immunol. 2018 May 30;9:1125. doi: 10.3389/fimmu.2018.01125 (PMC5992387; doi:10.3389/fimmu.2018.01125)
Supplement: Supplementary file 1 [file Image_1.PDF]

Supplement

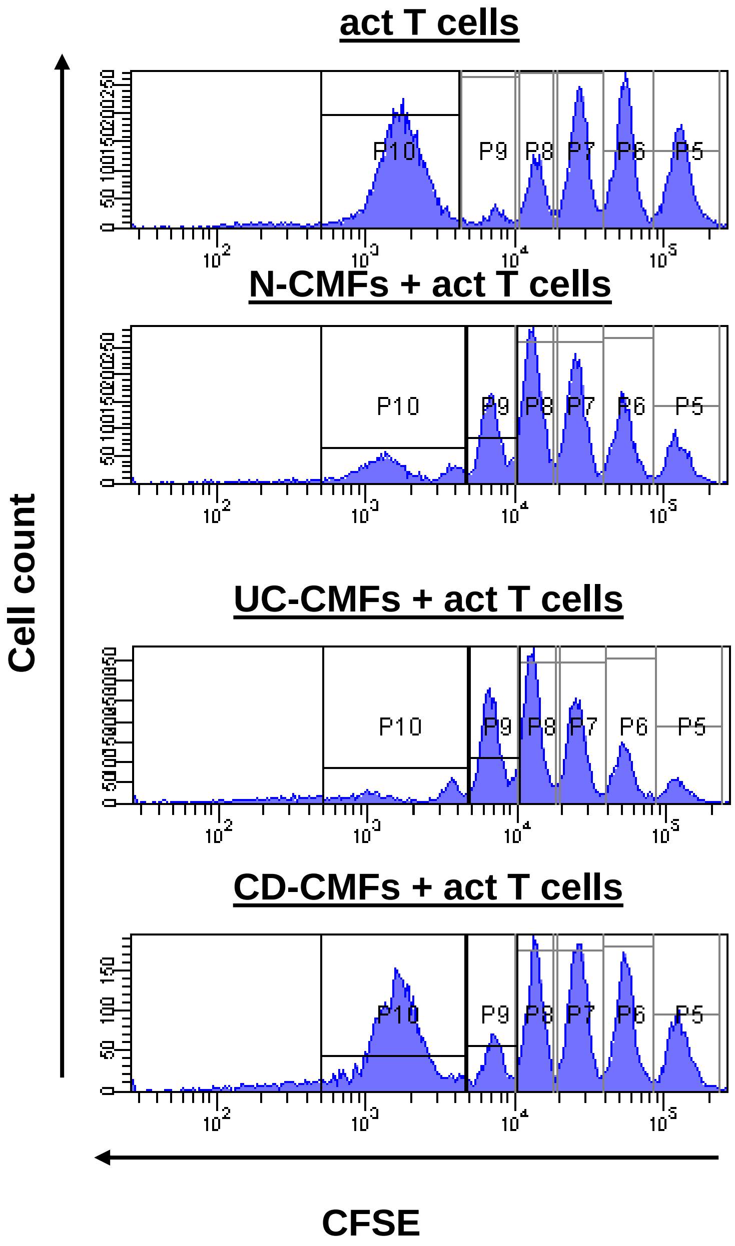

Fig. S1. When compared to N-CMFs, UC-CMFs demonstrated stronger suppression of the CD3/CD28 activated $CD4^+$ T cell proliferation, while CD-CMF-mediated suppression was significantly reduced. CMFs co-cultured with allogeneic CFSE-labeled, CD2/CD3/CD28-preactivated naive $CD4^+$ T cells at ratio 1:2.5 for 5 days in 24-well plates. T cell were then isolated from co-cultures, separated from magnetic beads and analyzed by flow cytometry. A representative experiment is shown, n=4 allogeneic donors per group.
